# Intranasal delivery of nanomicelle curcumin promotes corneal epithelial wound healing in streptozotocin-induced diabetic mice

**DOI:** 10.1038/srep29753

**Published:** 2016-07-11

**Authors:** Chuanlong Guo, Mengshuang Li, Xia Qi, Guiming Lin, Fenghua Cui, Fengjie Li, Xianggen Wu

**Affiliations:** 1 State Key Laboratory Cultivation Base, Shandong Provincial Key Laboratory of Ophthalmology, Shandong Eye Institute, Shandong Academy of Medical Sciences, Qingdao 266071, China; 2 School of Medicine and Life Sciences, Shandong Academy of Medical Sciences, University of Jinan, Jinan 250022, China; 3 Department of Pharmacy, College of Chemical Engineering, Qingdao University of Science and Technology, Qingdao 266042, China

## Abstract

Corneal nerves are mainly derived from the ophthalmic branch of the trigeminal ganglion (TG). Corneal neuropathy contributes to epithelial degenerative changes in diabetic keratopathy. Efficient drug delivery to TG may be beneficial for the treatment of diabetic keratopathy. This article described intranasal delivery of nanomicelle curcumin to correct pathophysiological conditions in TG to promote corneal epithelial/nerve wound healing in streptozotocin-induced diabetic mice. A diabetic mice model with corneal epithelium abrasion was established. Ocular topical and/or intranasal nanomicelle curcumin treatments were performed, and treatment efficacy and mechanisms of action were explored. Results showed that intranasal nanomicelle curcumin treatment promoted corneal epithelial wound healing and recovery of corneal sensation. Enhanced accumulation of reactive oxygen species, reduced free radical scavengers, increased mRNA expressions of inflammatory cytokines, and decreased mRNA expressions of neurotrophic factors in the cornea and TG neuron were observed in diabetic mice with corneal epithelium abrasions. Intranasal nanomicelle curcumin treatment effectively recovered these pathophysiological conditions, especially that of the TG neuron, and a strengthened recovery was observed with ocular topical combined with intranasal treatment. These findings indicated that intranasal curcumin treatment effectively helped promote diabetic corneal epithelial/nerve wound healing. This novel treatment might be a promising strengthened therapy for diabetic keratopathy.

Though diabetic retinopathy is the major diabetic complication in the eye, other parts of the eye are also affected^1^. Diabetes increases the risk of corneal abnormalities including recurrent erosions, delayed and incomplete wound healing, ulcers and edema, complications after vitrectomy, laser photocoagulation, and corneal surgery, as well as limbal epithelial stem cell alterations. For example, Schultz *et al*. reported that 47–64% of diabetic patients suffered from keratopathies^2^. Report showed that as high as 73.6% of the patients with diabetes had corneal complications, including punctate keratopathy, pannus, endothelial dystrophy, and corneal ulcer^3^. Corneal neuropathy, manifested by loss of corneal sensation and progressively reduced density of corneal stromal and subbasal nerves is also widespread in diabetic patients. Diabetic neuropathy contributes to epithelial degenerative changes seen in diabetic keratopathy. In animal models, diminished sensation and corneal nerve changes appear within the first 1 to 2 months of diabetes, which supports the role of neuropathy in causing keratopathy^4^.

Diabetic keratopathy is currently treated with lubricants and antibiotics, bandage contact lens, and tarsorrhaphy, all of which try to create a more favorable environment for wound healing^5^. However, even in combination, these measures may be ineffective and inadequate at accelerating re-epithelialization in diabetes^5^. The failure of conventional methods in corneal epithelial wound repair are not just related to pain and inconvenience, but also provide a window for infection that can lead to devastating and irreparable vision problems. Moreover, none of the present therapies address the fundamental pathobiology of delayed corneal healing secondary to diabetes. Therefore, novel methods are desired for the treatment of this tough complication of diabetes^6^.

Corneal nerves are mainly derived from the ophthalmic branch of the trigeminal nerve^7^. The cornea is the most innervated tissue in the entire human body (400 times more than the skin)^8^. Corneal innervation plays a critical role in protecting the cornea from mechanical, chemical, and thermal stimuli, as well as in producing trophic factors that are necessary for the maintenance of a healthy ocular surface. Decreased corneal innervation and sensitivity in diabetic patients leads to impaired epithelial wound healing, predisposing patients to sight-threatening complications such as stromal opacification, surface irregularity, and microbial infection. Decreased corneal sensation with a loss of innervation is one of the main causes of corneal complications in diabetic patients^9^. However, the exact mechanisms and interactions between trigeminal nerves and corneal cells remain unclear. It is known that nerve-secreted neuropeptides influence corneal cell proliferation *in vitro* and that corneal epithelial cell mitosis is altered in denervated rats^8^. And, a diabetes-associated depletion of neuropeptides in the trigeminal ganglion (TG) has already been confirmed. Diabetic corneal abnormalities may reflect a loss of trophic influences from the trigeminal nerve^10^. So, target delivery to the trigeminal nerve may be beneficial for the treatment of the diabetic keratopathy. However, there are no reports on how to target the applied drugs to the trigeminal nerve.

Studies in the past few years have shown intranasal drug delivery along the trigeminal nerve pathway. Throne *et al*. found about 10 folds higher radioactivity in the trigeminal nerve branches than in the olfactory bulb after intranasal administration of ^125^I-IGF-I^11^. Evidence suggests that an intranasally administered drug can reach the trigeminal nerve and perineural space from the absorbent respiratory and olfactory pseudoepithelium, because they are innervated by the trigeminal nerve. In addition, the trigeminal nerve covers and travels through the maxillary sinus, which is connected to the nasal cavity and is also lined by a thin pseudoepithelium. As the trigeminal nerve can transport a drug to the forebrain and hindbrain and other connected structures, trigeminally innervated structures such as the eye may also receive drug from the trigeminal nerve. Despite the possibility of a drug being transported to the brain, treatment of the trigeminal nerve without affecting the brain may be possible, because the trigeminal nerve showed a 20-fold higher drug concentration than the brain^12^.

Curcumin is a yellow-colored bioactive constituent of the perennial plant*, Curcuma longa* L., which possesses a wide range of physiological and pharmacological properties such as antioxidant, anti-inflammatory, anticancer, neuroprotective^13^. Scientific literature also shows that curcumin possesses anti-diabetic effects and mitigates diabetes complications owing to its potent ability to suppress oxidative stress and inflammation^13^. However, a major limitation with curcumin use is its low bioavailability^14^. Curcumin has also been proven effective for ophthalmic use in various ocular pathologies. However, its main drawbacks such as low solubility, instability, poor bioavailability limit the clinical application of curcumin in ophthalmology^15^. Current trends in curcumin research have concentrated on the development of potential delivery systems (such as nanocarrier delivery system) to increase its aqueous solubility, stability, and bioavailability as well as delivery at or around target tissues.

Recent studies show that polymeric micelle is one of the most attractive nanocarriers for hydrophobic drugs and its use improves drug’s bioavailability^16^,^17^. Amphiphilic block copolymers can form nano-sized aggregates with core-shell structures, which can solubilize poorly water-soluble drugs. Polymeric nanomicelles based on a new amphiphilic polymer polyvinyl caprolactam-polyvinyl acetate-polyethylene glycol graft copolymer (PVCL-PVA-PEG, Soluplus^®^) is an attractive strategy for improving the corneal bioavailability of therapeutic drugs while showing excellent ocular tolerance^16^; it may also be a promising strategy for drug delivery through intranasal administration without any modification to the drug molecule.

In this study, we intranasally administrated a nanomicelle curcumin formulation based on PVCL-PVA-PEG to evaluate the drug’s efficacy in experimental diabetic corneal epithelial/nerve wound healing. We also sought to demonstrate that the TG was involved in the mechanism of intranasal administration of nanomicelle curcumin against diabetic corneal epithelial/nerve wound healing.

## Results

### Nanomicelle curcumin promotes corneal epithelial wound healing and sensitivity recovery in diabetic mice

**Figure 1:**
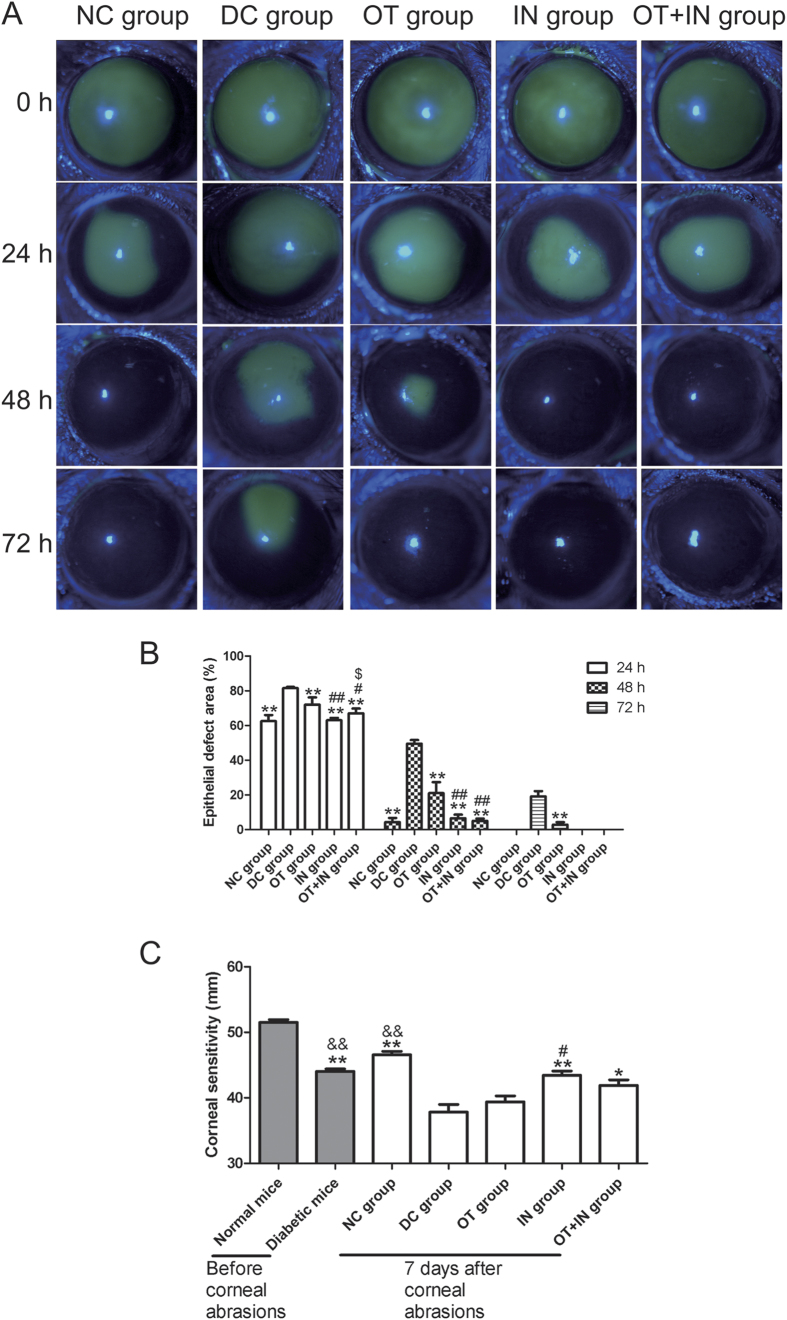
Nanomicelle curcumin promotes corneal epithelial wound healing and sensitivity recovery in diabetic mice. The effect of 7-day treatment with topical and/or intranasal nanomicelle curcumin in promoting diabetic corneal epithelial wound healing after corneal epithelium abrasion was investigated. (**A**) The corneal epithelial healing rate was significantly different from 24 h after corneal epithelium abrasion. (**B**) The corneal epithelial defect significantly decreased in diabetic mice treated with nanomicelle curcumin when compared with the DC group mice, and the decrease was similar to that in the NC group mice (**P < 0.01 when compared with the DC group; ^#^*P* < 0.05, with the OT group; ^##^*P* < 0.01, with the OT group; ^$^*P* < 0.05, with the IN group; n = 6). (**C**) The attenuated corneal sensitivity in diabetic mice recovered in the nanomicelle curcumin treatment groups (*P < 0.05 when compared with the DC group; **P < 0.01, with the DC group; ^#^*P* < 0.05, with the OT group; ^##^*P* < 0.01 with the OT group; ^&&^*P* < 0.01, with normal mice before corneal epithelium abrasion; n = 6).

The effect of topical nanomicelle curcumin solution in promoting diabetic corneal epithelial wound healing after the abrasion of corneal epithelium was examined. As shown in Fig. 1A,B, the corneal epithelial healing rate was significantly different from 24 h after corneal epithelium abrasion. The corneal epithelium defect in the ocular topical treatment group (OT group) mice (24 h: 72.11 ± 4.24%; 48 h: 21.07 ± 6.36%; 72 h: 2.92 ± 1.35%) significantly decreased when compared with the defect size in the diabetic control group (DC group) mice (24 h: 81.50 ± 0.71%; 48 h: 49.50 ± 2.12%; 72 h: 19.09 ± 3.13%). Moreover and interestingly, a significantly different and better corneal epithelial healing was observed in the intranasal treatment group (IN group) mice (24 h: 63.00 ± 1.41%; 48 h: 6.50 ± 2.12%). A strengthened effect was observed in the ocular topical combined with intranasal treatment group (OT + IN group) mice (24 h: 67.00 ± 2.82%; 48 h: 5.02 ± 1.44%), and the epithelial healing rate in the IN and OT + IN groups was similar to that in the normal control group (NC group) mice (24 h: 62.5 ± 3.54%; 48 h: 4.25 ± 2.47%), as all the corneas were completely healed in these two nanomicelle curcumin-treated groups at 72 h. Figure 1C shows the corneal sensitivity before epithelium abrasion and at 7 days after nanomicelle curcumin treatment. The corneal sensitivity was significantly attenuated in diabetic mice when compared with normal mice (*P* < 0.01), and was further attenuated after the corneal epithelium abrasions (*P* < 0.01), although the cornea recovered 7 days after nanomicelle curcumin treatment. The attenuated corneal sensitivity in the OT group showed non-significant improvement when compared with that in the DC group (*P* ≥ 0.05). In contrast, better corneal sensitivity recovery was noted in the IN group when compared with the DC group (*P* < 0.01). However, there was not a strengthened effect for corneal sensitivity observed in the OT + IN group (*P* ≥ 0.05 when compared with the IN group).

**Figure 2:**
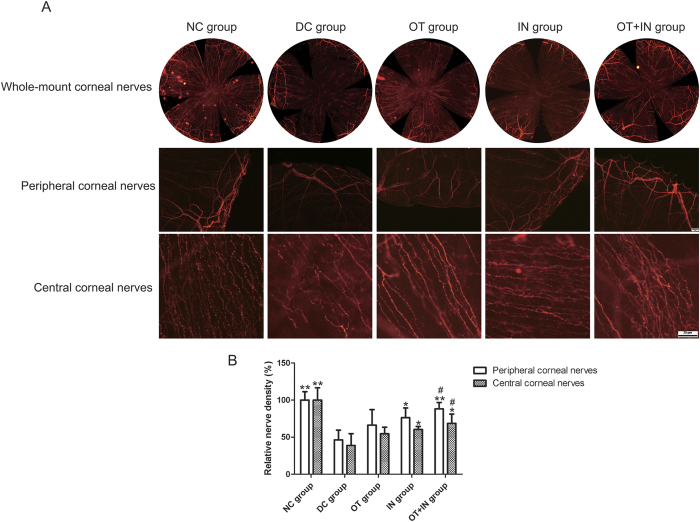
Nanomicelle curcumin accelerates corneal nerve regeneration in diabetic mice. The effect of 7-day treatment with topical and/or intranasal nanomicelle curcumin in promoting diabetic corneal nerve regeneration after corneal epithelium abrasion was investigated. Corneas were harvested, flat-mounted, and immunostained with β-III tubulin antibody. (**A**) Images of central and peripheral corneal nerve were taken and combined as a whole flat-mounted corneal nerve. (**B**) Central and peripheral corneal nerve densities were calculated and are expressed as a percentage of that in the NC group (*P < 0.05 when compared with the DC group; **P < 0.01, with the DC group; ^#^*P* < 0.05, with the OT group; n = 3).

The effects of nanomicelle curcumin on corneal epithelial nerve regeneration were further characterized. One week after debridement, corneal nerve densities in the DC group were found to be significantly different from that in the NC group (Fig. 2A). Compared with the DC group, the OT group showed no significant improvement in both peripheral and central corneal nerve densities (*P* ≥ 0.05); however, the IN group and OT + IN group mice showed significant improvement, and a strengthened effect was observed in the OT + IN group (*P* < 0.05 when compared with the OT group; Fig. 2B). These results suggest that in diabetic mice, intranasal nanomicelle curcumin solution promotes corneal epithelial wound healing accompanied with recovery of corneal sensitivity and corneal nerve density.

### Nanomicelle curcumin decreases proinflammatory cytokine expression in cornea and TG neuron in diabetic mice

**Figure 3:**
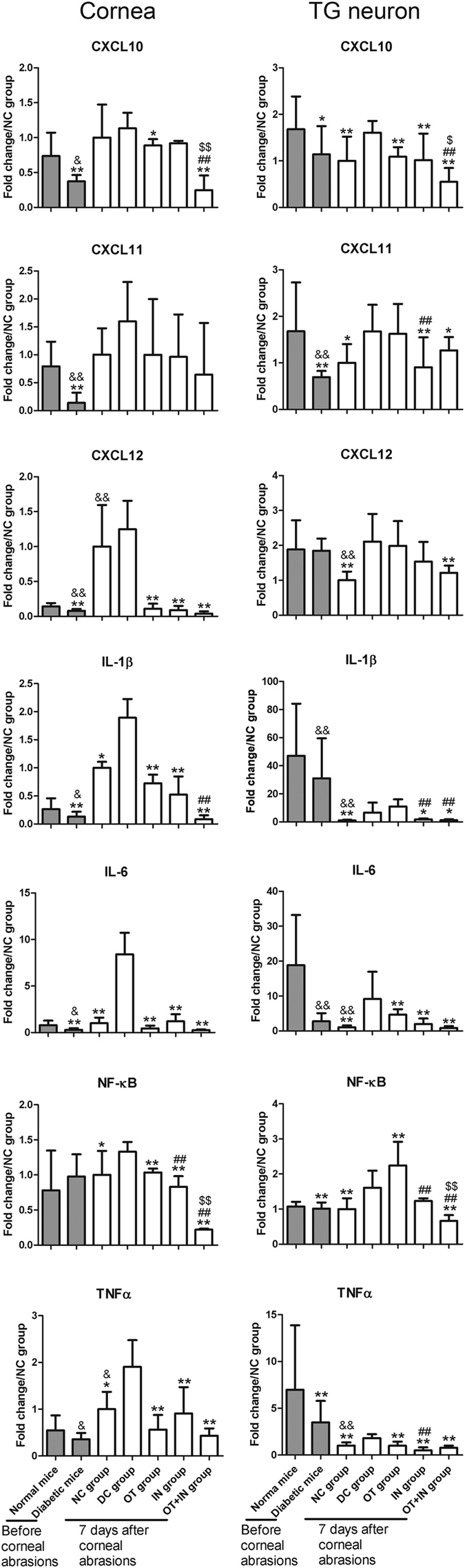
Proinflammatory cytokine mRNA expression in the cornea and TG neuron in age-matched normal and diabetic mice, and in normal and diabetic mice after corneal epithelium abrasion and 7-day nanomicelle curcumin solution treatment. Data are expressed as mean ± SD (*P < 0.05 when compared with the DC group; **P < 0.01, with the DC group; ^#^*P* < 0.05, with the OT group; ^##^*P* < 0.01, with the OT group; ^$^*P* < 0.05, with the IN group; ^$$^*P* < 0.01, with the IN group; ^&&^*P* < 0.01, with the age-matched normal mice without corneal epithelium abrasion; n = 4).

After observation of corneal epithelial wound/nerve healing with nanomicelle curcumin treatment in the diabetic mice model with corneal epithelium abrasion, whether this effect was associated with altered expression of proinflammatory cytokines was further explored (Fig. 3). For exploring the changes in the expression profiles of proinflammatory cytokines, the cornea and TG neuron from age-matched normal and diabetic mice without corneal epithelium abrasions were also analyzed. Compared with normal mice without corneal epithelium abrasion, diabetic mice without corneal epithelium abrasion showed significantly decreased expressions of proinflammatory cytokines in the cornea (CXCL10/11/12, IL-1β, IL-6, and TNFα: *P* < 0.05 or 0.01) or showed no significant changes (NF-κB, *P* ≥ 0.05). In contrast, after corneal epithelium abrasion, the expressions of proinflammatory cytokines in the cornea were increased in both the normal control and diabetic mice when compared with mice without abrasion, although the corneal epithelium abrasion had completely healed, as observed through slit lamp observation at 7 days after abrasion. Among groups with corneal epithelium abrasion, proinflammatory cytokines such as IL-1β, IL-6, NF-κB, and TNFα were significantly increased in the DC group than in the NC group (*P* < 0.05 or 0.01). CXCL10, CXCL11, and CXCL12 were not observed with significantly increased (*P* ≥ 0.05). All cytokines other than CXCL11 were decreased following nanomicelle curcumin treatment. Regarding different treatment groups, interesting findings were the significant decrease of NF-κB in the IN group when compared with the OT group (*P* < 0.01), and the strengthened effect in the OT + IN group for the decrease of CXCL10, IL-1β, and NF-κB.

Regarding changes in the TG neuron in normal and diabetic mice without corneal epithelium abrasion, the findings were similar to those for the cornea. After nanomicelle curcumin treatment, a significant decrease of CXCL10, IL-6, and TNFα; no significant regulation of CXCL11, CXCL12, and IL-1β; but increase of NF-κB were observed in the OT group when compared with the DC group. However, all of these tested proinflammatory cytokines except for CXCL12 and NF-κB showed a significant decrease in the IN group when compared with the DC group (*P* < 0.05 or 0.01). Moreover, all eight proinflammatory cytokines were significantly decreased in the OT + IN group (*P* < 0.05 or 0.01), indicating a strengthened effect of the ocular topical and intranasal nanomicelle curcumin solution treatment.

**Figure 4:**
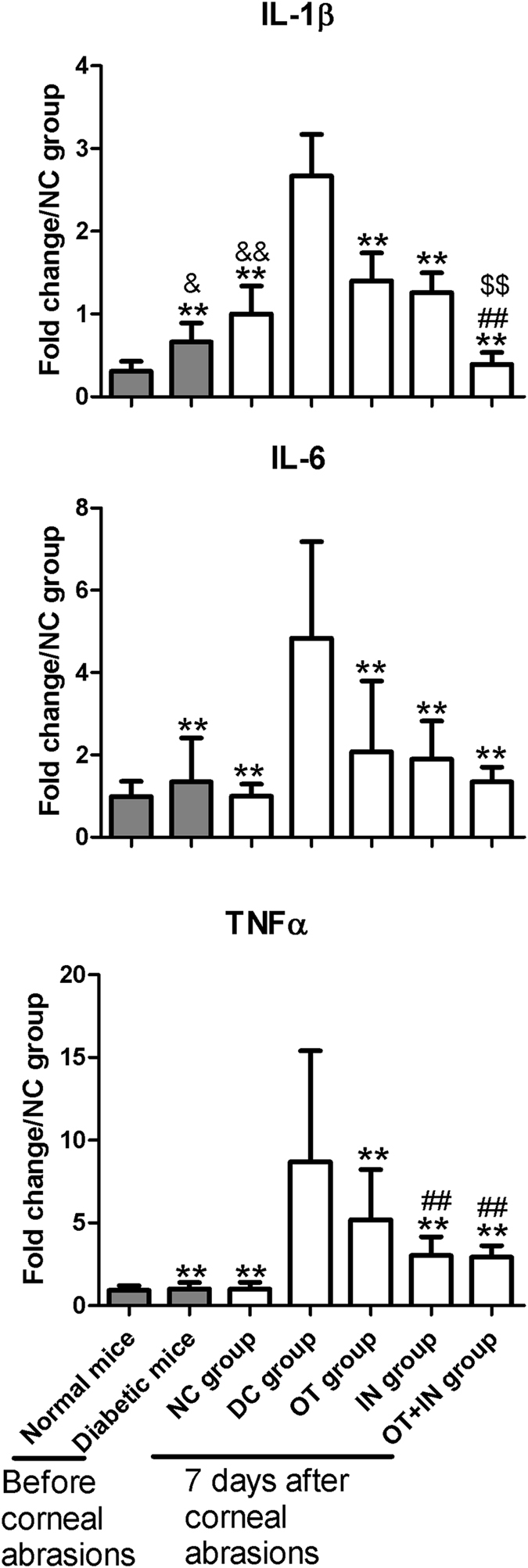
Proinflammatory cytokine mRNA expression in the brain in normal and diabetic mice before and after corneal epithelium abrasion and 7-day nanomicelle curcumin treatment. Data are expressed as mean ± SD (*P < 0.05 when compared with the DC group; **P < 0.01, with the DC group; ^#^*P* < 0.05, with the OT group; ^##^*P* < 0.01, with the OT group; ^$^*P* < 0.05, with the IN group; ^$$^*P* < 0.01, with the IN group; ^&&^*P* < 0.01, with the normal mice without corneal epithelium abrasion; n = 4).

As to the regulation profiles of the inflammatory brain biomarkers, diabetic mice without abrasion showed IL-1β expression increased or IL-6 and TNFα expression with non-significantly changed when compared with normal mice without corneal epithelium abrasion. In the DC group, similar to the cornea and TG neuron tissues, corneal epithelium abrasions induced a strong increase of these proinflammatory cytokines in the brain when compared with the NC group (*P* < 0.01). Nanomicelle curcumin administered in all three protocols produced a significant decrease of proinflammatory cytokines, and a strengthened effect was observed for IL-1β and TNFα in the OT + IN group (Fig. 4).

### Nanomicelle curcumin increases neurotrophic factor expression in cornea and TG neuron in diabetic mice

**Figure 5:**
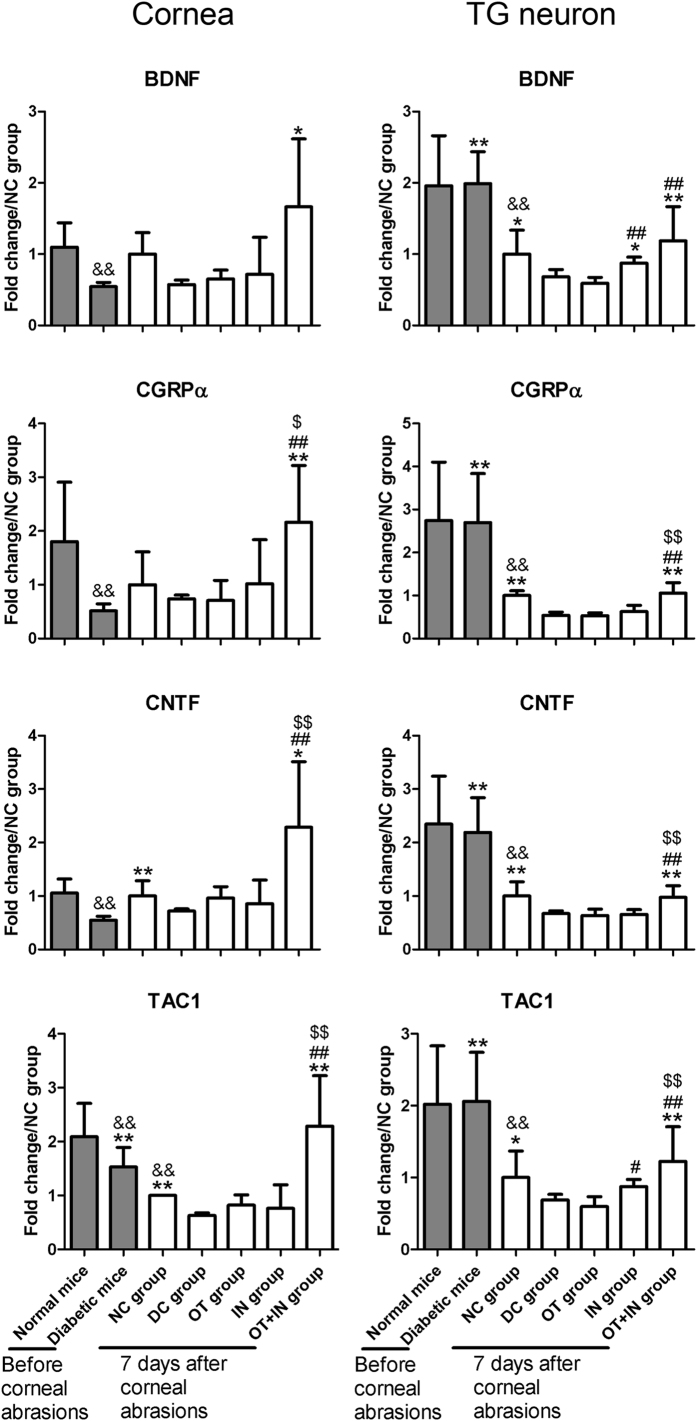
Neurotrophic cytokine mRNA expression in the cornea and TG neuron in age-matched normal and diabetic mice with no corneal epithelium abrasion, and normal and diabetic mice with corneal epithelium abrasion and 7-day nanomicelle curcumin solution treatment. Data are expressed as mean ± SD (*P < 0.05 when compared with the DC group; **P < 0.01, with the DC group; ^#^*P* < 0.05, with the OT group; ^##^*P* < 0.01, with the OT group; ^$^*P* < 0.05, with the IN group; ^$$^*P* < 0.01, with the IN group; ^&&^*P* < 0.01 with the age-matched normal mice without corneal epithelium abrasion; n = 4).

Similar to proinflammatory cytokines, changes in the expression profiles of neurotrophic factors in the cornea and TG neuron in age-matched normal and diabetic mice without corneal epithelium abrasions were also analyzed. Compared with normal mice without corneal epithelium abrasion, age-matched diabetic mice without corneal epithelium abrasion showed significantly decreased expression of all neurotrophic factors in cornea. However, after corneal epithelium abrasion, the expression of all these neurotrophic factors except for TAC1 (the gene encoding substance P, SP) in the cornea showed no significant regulation in both NC group and DC group when compared with their counterparts with no abrasion. When comparing the groups with corneal epithelium abrasion, abrasions induced a strong decrease of neurotrophic factors in the cornea in the DC group when compared with the NC group. In particular, neurotrophic factors such as ciliary neurotrophic factor (CNTF) and TAC1 were significantly decreased (*P* < 0.05 or 0.01), whereas brain-derived neurotrophic factor (BDNF) and calcitonin gene-related peptide (CGRP) α isoform (CGRPα) showed a non-significant decrease (*P* ≥ 0.05). These cytokines were increased in the OT + IN group, though none of the five neurotrophic factors showed significant regulation in the OT group and IN group (*P* ≥ 0.05; Fig. 5).

Regarding the TG neuron, the expressions of all neurotrophic factors in diabetic mice without corneal epithelium abrasion were similar to those in age-matched normal mice without corneal epithelium abrasion. However, after corneal epithelium abrasion, the expressions of all neurotrophic factors in the cornea were significantly decreased in the TG neuron in both the NC group and DC group when compared with their counterparts without abrasion (*P* < 0.01). When comparing the groups with corneal epithelium abrasion, the DC group showed significant decrease of all four neurotrophic factors when compared with the NC group (*P* < 0.05 or 0.01). No significant regulations were observed in the OT group (*P* ≥ 0.05), while significant regulation of BDNF was observed in the IN group (*P* < 0.05). Interestingly, a strengthened effect was also observed in the OT + IN group, where all neurotrophic factors were significantly increased, when compared with the DC group (*P* < 0.01; Fig. 5).

**Figure 6:**
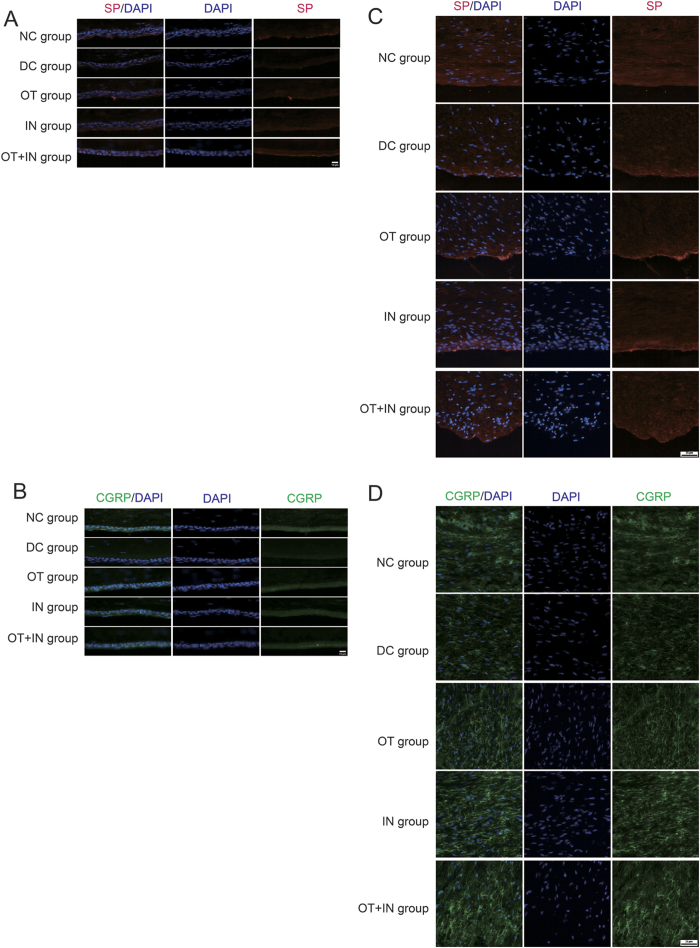
Nanomicelle curcumin treatment increases expressions of two main neurotrophic cytokines in the cornea and TG neuron in diabetic mice. Immunoflurorescent staining shows that nanomicelle curcumin treatment restored the protein levels of SP and CGRP in the cornea and TG neuron. (**A**) Images of SP in the cornea (n = 3). (**B**) Images of CGRP in the cornea (n = 3). (**C**) Images of SP in the TG neuron (n = 3). (**D**) Images of CGRP in the TG neuron (n = 3). The bar on corneal images is 10 μm, and that on TG images is 25 μm.

SP (Fig. 6A) and CGRP (Fig. 6B) were also tested with immunofluorescence in mice with corneal epithelium abrasion, and the results thus obtained were in agreement with the PCR results. SP and CGRP were found to be predominant in the basal cell layer of the corneal epithelium, and their expressions were decreased in the DC group when compared with the NC group. The fluorescence intensity improved in the nanomicelle curcumin treatment groups, especially in the OT + IN group, where the SP expression recovered and the fluorescence intensity was higher than that in the DC group. Regarding the TG neuron findings, SP (Fig. 6C) and CGRP (Fig. 6D) were found in the whole tissue, and the changes in their expression profiles were similar to those in the cornea.

## Discussion

Corneal innervation and the resulting pathologies associated with denervation have been the topic of research for a long time. Investigators have examined the effects of neuropathy and shown that diabetes-induced denervation of the cornea reduces the viability of the corneal epithelium and its ability to repair damage^18^. However, the fate of TG neuron in diabetic pathological condition remains unrevealed. But there exist some indirect evidences for its pathological condition^10^. In this study, we found that the mRNA expressions of some inflammatory cytokines in the cornea and TG neuron of diabetic mice with corneal epithelium abrasion were significantly lower than their normal counterparts (Fig. 3), and a similar observation was also made for the mRNA expression of some neurotrophic factors (Fig. 5). However, normal and diabetic mice with corneal epithelium abrasion produced different results. We observed an imbalance in the redox state and disorders in inflammatory cytokines and neurotrophic factor mRNA expression in the TG neuron and in the cornea in diabetic mice 7 days after corneal epithelium abrasion (Fig. S1, Figs 3 and 5). Enhanced accumulation of ROS and reduced free radical scavengers were observed in both the corneal epithelium and the TG neuron in diabetic mice when compared with normal mice, and findings of the cornea were consistent with previous reports^19^. Increased mRNA expression of proinflammatory cytokines and decreased mRNA expression of neurotrophic factors were observed both in the cornea and TG neuron, and findings of the cornea were also consistent with previous reports^20^. These findings suggest that corneal epithelium abrasion triggered the responses of inflammatory cytokines and neurotrophic factors in the cornea and TG neuron, and that the TG neuron, as well as the innervation of the cornea, was impaired in diabetic mice.

The corneal-TG axis is represented anatomically by the trigeminal/corneal nerves and functionally by specific neurotransmitters such as SP^21^. Neuropeptides secreted from the TG neuron regulate the proliferation of corneal cells, and contribute to establishing and maintaining the corneal structure and function^22^. Surgical axotomy of the ciliary nerve of the trigeminal nerve in adult BALB/c mice at the posterior sclera caused neurotrophic keratopathy^21^. The expression of TAC1 and TAC1R (coding for the SP receptor, NK-1R) could be altered in the TG neuron following chemical burn of the cornea^21^, as most of corneal neurons locate in the ophthalmic division of the TG neuron^23^. Oxidative stress and inflammation play a pivotal role in diabetes and its complications^24^,^25^. As the TG, as well as the innervation of the cornea, was impaired in diabetic mice, we hypothesized that correcting the imbalance in the redox state and restoring disorders of inflammatory cytokine and neurotrophic factor expression in the TG neuron would help corneal re-innervation and re-epithelialization in diabetes.

The question that arises is how to target therapy such as curcumin to the pathological TG? Though local TG injection treatment has been reported previously^26^,^27^, delivering enough medical agents to the TG remains a challenge. However, intranasal drug delivery targets the TG by using the trigeminal nerve pathway, as well as other associated structures, as a conduit to transport a drug. More importantly, such a targeted delivery may be possible even without affecting the connected structures, because a higher drug concentration has been reported in the trigeminal nerve than in the connected structures such as the brain (20-fold)^12^.

To further test the hypothesis mentioned above, an intranasal delivery of nanomicelle curcumin solution was performed to study its effectiveness in healing corneal/nerve lesions in diabetic mice, and the results thus obtained were indeed inspiring(Figs 1 and 2). Oxidative stress and inflammation are involved in the development and progression of diabetes and its complications^28^. Some neurotrophins, neurotrophic factors and nerve guidance factors, such as BDNF, CGRP, CNTF, and SP, are important to corneal nerve regeneration^10^,^29^,^30^. SP and CGRP constitute the main sensory peptides in the TG neuron, and these neuropeptides are also the major sensory neurotransmitter and neuropeptide released from corneal nerve fibers^10^,^31^. Diabetic abnormalities may reflect a loss of trophic influences from the trigeminal nerve and cornea, as both SP and CGRP exert trophic influences on various peripheral tissues and cell types. While curcumin is a well known antioxidant and anti-inflammatory agent, and it also has neurotrophic activity^32^. So, the effect on ROS, proinflammatory cytokine and neurotrophic factor expression changes in cornea and TG neuron in diabetic mice after nanomicelle curcumin treatment with different administration route were investigated.

The effect of nanomicelle curcumin on the regulation of diabetes-induced oxidative stress in the corneal epithelium and TG neuron was evaluated by the detection of intracellular ROS. It was interestedly found that the imbalance in the redox state in the cornea and TG neuron was significantly corrected in the IN group, as well as in the OT group. While the intranasal treatment, compared with the ocular topical administration treatment, showed greater regulation of mRNA expression of inflammatory cytokines and neurotrophic factors in the TG neuron. We could safely conclude that the curcumin concentration in the cornea in the OT group was much higher than that in the IN group, while exciting results of the corneal epithelial wound healing and sensitivity recovery in the IN group were observed, and some observations were even much better than those in the OT group. The events underlying corneal re-epithelialization and sensitivity recovery were complex, but could be partly attributed to the regulation profiles of ROS and free radical scavengers, and the mRNA expressions of inflammatory cytokines and neurotrophic factors in the TG neuron, as well as the cornea.

Similarly to intranasal delivery, ocular delivery is also as a possible approach to deliver a drug to the TG and brain, and it is possible that both ocular and intranasal delivery routes may involve the trigeminal pathway^33^. However, the delivery efficiency may differ. In our previous experiments, the concentration of model agent coumarin-6 in the TG neuron was determined after intranasal or ocular topical delivery of coumarin-6 nanomicelle solution (0.5 mg/ml) with similar method as reported^12^,^34^; the concentrations of coumarin-6 in the TG neuron after intranasal delivery were 138.3%, 96.9%, and 40.7% higher than those after ocular topical delivery at 15, 60, and 120 min after the administration, respectively (unpublished data). These results imply that the intranasal route may be more efficient than the ocular topical approach in delivering the drug to the TG neuron. Coumarin-6 was also detected in the cornea after intranasal delivery, but the concentration was only 0.7%, 2.2%, and 92.9% of the corneal concentration after ocular topical delivery at 15, 60, and 120 min after the administration, respectively. Our recent experiment results confirmed the high overlap of coumarin-6 in the TG neurons after ocular topical or intranasal administration using the the Fluoro-Gold™ nerve tracing tests, this results further confirmed the trigeminal ganglion - corneal nerve transporting pathway. Although, in this study, we did not obtain direct concentration of the curcumin nanomicelle solution, but the confocal laser scanning microscopy observation results were similar to those of the coumarin-6 nanomicelle solution. Therefore, we could speculate that it was the treatment of the TG that helped the recovery of corneal epithelium and re-innervation of corneal nerve. Our results are in agreement with previous reports^33^,^35^,^36^. Further more, weaker dynamics of the retrograde transport of horseradish peroxidase (HRP) in the trigeminal nerve during burn keratitis was associated with the damaging effect of high temperature and inflammation on HRP uptake and transportation^37^. In our study, the corneal epithelium was scraped and nerve fibers were partially hampered; this might have also contributed to the attenuated treatment efficacy at the TG neuron after ocular administration, while this corneal epithelium/nerve fibers hamper did not exert influence to intranasal administration.

Medical agents could be delivered to the brain via the intranasal route, though the concentration was much lower than that in the TG neuron. Special attention should be paid to brain toxicity effects of a drug after intranasal delivery. In the intranasal drug delivery system, brain toxicity was usually assessed based on apoptosis assay and tumor necrosis factor for inflammation^38^,^39^. The regulation profiles of the inflammatory brain biomarkers—IL-1β, IL-6, and TNFα—were analyzed to explore how nanomicelle curcumin solution treatment affected the brain tissues^38^. We found disorders of inflammatory cytokines in diabetic mice brain, and these results are similar to those reported previously^40^. However, not only brain toxicity was absent but the disorders of inflammatory cytokines in brain were also better corrected in animals that received intranasal drug as compared with ocular topical delivery. The TUNEL staining results also suggested that nanomicelle curcumin did not have negative effects in normal and diabetic brain (Fig. S2). The results suggest that intranasal nanomicelle curcumin solution does not have any negative affect; rather it produces positive effects in the diabetic brain.

We however need to pay special attention to nasal ciliotoxicity when considering intranasal administration of the curcumin nanomicelle solution. From the *in situ* toad palate model and *in vivo* mice nasal mucosa model, it can be safely concluded that nanomicelle curcumin solution showed no toxicity on nasal mucosa and is therefore safe for nasal administration (Fig. S3). PVCL-PVA-PEG is safe as an ocular drug delivery system^16^, and our previous experiment has shown that the nanomicelle curcumin solution was safe for topical ocular application (unpublished data). To our knowledge, this study is the first to explore the use of PVCL-PVA-PEG as a nasal drug delivery system, and the safety of PVCL-PVA-PEG as a nasal drug delivery system would expand this polymer and its drug delivery system’s administration route.

Although the study afforded promising results, there are not enough studies yet and further investigation is warranted. One limitation is that the untreated and treated control groups for both diabetic and normal mice with corneal epithelium abrasions are not yet adequately designed. In our preliminary test, we realized that well-designed, consistent corneal epithelium abrasions are crucial to analyze the results; therefore, in this study, all the corneal epithelium abrasions were created by one investigator (M.L.). However, if fully designed of untreated control groups and treated control groups (for example, untreated normal control group, normal control group with PBS ocular topical treatment, normal control group with PBS intranasal treatment, untreated diabetic control group, diabetic control group with PBS ocular topical treatment, diabetic control group with PBS intranasal treatment), the so long whole operation procedure duration lasted could result skewed results. Therefore, after confirming that the results are not skewed among the untreated and treated control group in diabetic/normal mice with small samples of corneal healing in a preliminary test, we performed experiments with the NC group and DC group. Another limitation is that because of limited samples (the shortage one also contributed to this), western blotting or ELISA detection could not be performed to further examine antioxidants/free radical scavengers, proinflammatory cytokines, and neurotrophic factors. Further study is needed to fully address these potential changing profiles during and after intranasal and/or ocular topical nanomicelle curcumin treatment.

## Conclusions

The findings of the present study indicate that intranasal delivery of nanomicelle curcumin solution promotes corneal epithelial/nerve healing in diabetic mice. Regarding the mechanism of action, intranasal treatment was effective in delivering the pharmaceutically active ingredient to the TG neuron, resulting in well treatment of TG, and this help diabetic corneal epithelial/nerve wound heal. To the best of our knowledge, this study is the first to explore the use of polymer PVCL-PVA-PEG-based nanomicelle as an ocular/nasal drug delivery system, and to explore the intranasal mode of administration to help improve the recovery of corneal epithelium lesion and corneal neuropathy in diabetes.

## Materials and Methods

### Animals

Details of the animal use and diabetic model information are described in the supporting information (SI) Materials and Methods. The animal care and procedures were conducted according to the Principles of Laboratory Animal Care. The use of animals in this study adhered to the ARVO Statement for the Use of Animals in Ophthalmic and Vision Research, and the animal study was approved by the Shandong Eye Institute Ethics Committee for Animal Experimentation (Approval document No 2012-6, Qingdao, Shandong, China).

### Corneal epithelial wound healing

**Figure 7:**
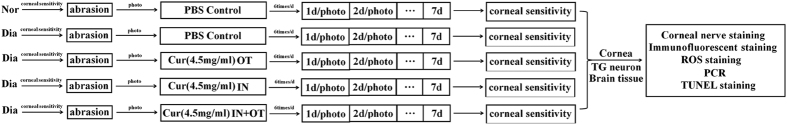
Experimental groups and protocols. Diabetic mice (12 weeks after induced as diabetic model), and the normal age-matched control mice were tested with corneal sensation, then received a central 2.5-mm diameter corneal epithelium abrasion. After epithelium abrasion, the eyes were intranasal and/or ocular topical treatment with PBS or nanomicelle curcumin for 7 consecutive days. The defects of corneal epithelium were visualized at 24, 48, and 72 h by instilling 0.25% fluorescein sodium and photographed under a slit lamp. Corneal sensation was re-tested 7 days after corneal abrasion. Then, the mice were sacrificed, corneas, TG neurons, and brains were carefully dissected, and further corneal nerve staining, immunofluorescent staining, real-time PCR, ROS generation and antioxidant molecule expression were further analyzed. Dia: diabetic mice; Nor: normal mice.

Details of corneal epithelial wound healing and treatments are described in the supporting information (SI) Materials and Methods, and the time course of the experiments is depicted in Fig. 7.

### Corneal sensitivity

Details of corneal sensitivity tests are described in the supporting information (SI) Materials and Methods.

### Corneal nerve staining

Corneal nerve staining was performed, as described previously, with an anti-neuron-specific β-III tubulin antibody (NL1195R; R&D System Inc. Minneapolis, USA). Details of corneal nerve staining are described in the supporting information (SI) Materials and Methods.

### Immunofluorescent staining

Corneas and TG neurons were snap-frozen in the Tissue-Tek optimum cutting temperature compound (OCT, Sakura Finetechnical, Tokyo, Japan). Details of immunofluorescent staining are described in the supporting information (SI) Materials and Methods.

### Reverse transcription quantitative-polymerase chain reaction (PCR)

Details of PCR procedures are described in the supporting information (SI) Materials and Methods.

### Measurement of ROS generation and antioxidant molecule expression

Details of ROS measurement and antioxidant molecule expression procedures are described in the supporting information (SI) Materials and Methods.

### Brain toxicity evaluation during the treatment

Details of brain toxicity evaluation procedures are described in the supporting information (SI) Materials and Methods.

### Nasal ciliotoxicity

Details of nasal ciliotoxicity evaluation procedures are described in the supporting information (SI) Materials and Methods.

### Statistical analysis

Data in this study are representative of more than three different experiments and presented as mean ± SD. The data were analyzed using SPSS software, version 11.5 (SPSS, Chicago, IL, USA). The significance of differences was evaluated using ANOVA with multiple comparisons. For all evaluations, a *P*-value less than 0.05 was considered statistically significant.

## Additional Information

**How to cite this article**: Guo, C. *et al*. Intranasal delivery of nanomicelle curcumin promotes corneal epithelial wound healing in streptozotocin-induced diabetic mice. *Sci. Rep.* **6**, 29753; doi: 10.1038/srep29753 (2016).

## Supplementary Material

Supplementary Information
