# Supplementary material for: Intranasal delivery of nanomicelle curcumin promotes corneal epithelial wound healing in streptozotocin-induced diabetic mice
Source: Sci Rep. 2016 Jul 11;6:29753. doi: 10.1038/srep29753 (PMC5378915; doi:10.1038/srep29753)
Supplement: Supplementary Information [file srep29753-s1.doc]

**Supporting Information (SI)**

**Intranasal delivery of nanomicelle curcumin promotes corneal epithelial wound healing in streptozotocin-induced diabetic mice**

**Running title:** Intranasal treatment for diabetic corneal wound

Chuanlong Guo^1,+^, Mengshuang Li^1,2,+^, Xia Qi^1^, Guiming Lin^1,2^, Fenghua Cui^1^, Fengjie Li^1^, Xianggen Wu^1,2,3*^

1 State Key Laboratory Cultivation Base, Shandong Provincial Key Laboratory of Ophthalmology, Shandong Eye Institute, Shandong Academy of Medical Sciences, Qingdao 266071, China; 2 School of Medicine and Life Sciences, Shandong Academy of Medical Sciences, University of Jinan, Jinan 250062, China; 3 Current affiliation: Department of Pharmacy, College of Chemical Engineering, Qingdao University of Science and Technology, Qingdao 266042, China

*Correspondence: [wuxianggen@126.com](mailto:wuxianggen@126.com)

+C. Guo and M. Li contributed equally to this article as the co-first authors.

**SI Materials and Methods**

**Animals**

Six-week-old male C57BL/6 mice were purchased from Beijing HFK Bioscience Co., Ltd. (Beijing, China; License No. SCXK [Jing] 2014-0004). All animals were healthy and free of clinically observable ocular abnormalities. The mouse care and procedures conformed to the Principles of Laboratory Animal Care, and the use of animals adhered to the ARVO Statement for the Use of Animals in Ophthalmic and Vision Research. This animal study was approved by the Shandong Eye Institute Ethics Committee for Animal Experimentation in Qingdao, Shandong, China. Type 1 diabetes mellitus was induced in mice by administering an intraperitoneal injection of 50 mg/kg streptozotocin (STZ; Sigma, St. Louis, MO) in ice-cold citrate-citric acid buffer (pH 4.5) for 5 consecutive days, while control mice received an equal amount of buffer. Before each injection, mice were fasted for 5 hours, and after each injection, mice were provided with 10% sucrose water to prevent sudden hypoglycemia. Blood glucose levels were monitored from the tail vein by using a OneTouch Basic glucometer (LifeScan, Johnson & Johnson, Milpitas, CA). Those mice with blood glucose level over 300 mg/dL by day 10 after the last STZ injection were considered as a diabetic model. All control mice remained normoglycemic. Mice were assessed for body weight and serum glucose level, and then assessed for the subsequent tests after 12 weeks of hyperglycemia, which corresponded to animal age of 20 weeks.

**Corneal epithelial wound healing**

Normal and diabetic mice were anesthetized by an intraperitoneal injection of xylazine (7 mg/kg) and ketamine (70 mg/kg) followed by topical application of 2% xylocaine. A central 2.5-mm diameter corneal epithelium was scraped with an algerbrush II corneal rust ring remover (Alger Co, Lago Vista, TX) and then one drop of levofloxacin eye drops was used immediately to avoid infection. Only one eye was wounded at a time in each animal. After corneal abrasion, normal mice were set as the normal control group (NC group) with 23-25 mice in this group: ocular topical combined with intranasal treatment with phosphate buffer solution (PBS). The diabetic mice were randomly distributed into four groups with 23-25 mice in each group: (1) diabetic control group (DC group): ocular topical combined with intranasal treatment with PBS; (2) ocular topical treatment group (OT group): ocular topical treatment with nanomicelle curcumin solution; (3) intranasal treatment group (IN group): intranasal treatment with nanomicelle curcumin solution; and (4) ocular topical combined with intranasal treatment group (OT+IN group): ocular topical combined with intranasal treatment with nanomicelle curcumin solution. For ocular topical and/or intranasal treatment, the drug was applied ocular topically and/or intranasally 6 times daily with 5 μl volume per eye/nostril dose for 7 days. For intranasal delivery, conscious mice were held on their back with the head in the upright position, as described previously^1^. The nanomicelle curcumin solution or PBS was administered intranasally to mice with a pipette, 5 μl per nostril, with a holding time of 1 min. The drop was placed at the opening nostril, allowing the mice to snort the drop into the nasal cavity. The nanomicelle curcumin solution was prepared and characterized, as described previously^2^. All of the solution used in this study was with a curcumin concentration of 4.5 mg/ml. The defects of corneal epithelium were visualized at 24, 48, and 72 h by instilling 0.25% fluorescein sodium and photographed under a slit lamp (BQ900; Haag-Streit, Bern, Switzerland). One drop of levofloxacin eye drops was used immediately after each fluorescein sodium staining to avoid infection. The staining area was analyzed by Image J software (National Institutes of Health, Bethesda, MD) and calculated as the percentage of residual epithelial defect. The time course of the experiments is depicted in Fig. 8.

**Corneal sensitivity**

Corneal sensation was tested before the corneal abrasion and 7 days after corneal abrasion. It was measured using a Cochet-Bonnet esthesiometer (Luneau Ophtalmologie, Chartres Cedex, France) in unanesthetized mice, as previously reported^3^. The testing began with a nylon filament of maximal length (6 cm), which was shortened by 0.5 cm each time until the corneal touch threshold was found. Each cornea was tested 4 times until there was no blink response. All measurements were performed by the same examiner.

**Corneal nerve staining**

Corneal nerve staining was performed, as described previously, with an anti-neuron-specific β-III tubulin antibody (NL1195R; R&D System Inc. Minneapolis, USA). The corneal nerves were counted using Image J software ^4,5^. Five image fields were sampled in each whole-mount corneal image, one at the center of the cornea and the rest midway between the central and limbal cornea for each of the corneal quadrants. Central and peripheral corneal nerve densities were calculated and expressed as a percentage of NC group^6^.

**Immunofluorescent staining**

Cornea and trigeminal ganglion (TG) neuron were snap-frozen in the Tissue-Tek optimum cutting temperature compound (OCT, Sakura Finetechnical, Tokyo, Japan). For immunofluorescent staining, cryosections were fixed by 4% paraformaldehyde, permeabilized with Triton X-100, and blocked with normal serum. The samples were stained with primary antibodies overnight at 4°C, and subsequently with fluorescein-conjugated secondary antibodies at 37°C for 1 h. All antibodies used are listed in Table S1. Negative control was obtained when the primary antibody was omitted from the incubation procedure. All staining was examined under a confocal microscope or an Eclipse TE2000-U microscope (Nikon, Japan) after counterstaining with 4’,6-diamidino-2-phenylindole (DAPI) or propidium iodide (PI).

Table S1 Antibody information

| Primary antibody | Supplier | Catalog number | Dilution (immunofluorescence) |
| --- | --- | --- | --- |
| Anti-neuron-specific β-Ⅲ Tubulin-NL-557 | R&D System Inc | NL1195R | 1:10 |
| Anti-NQO1 antibody | Abcam | Ab34173 | 1:200 |
| Anti-catalase antibody | Abcam | Ab16731 | 1:200 |
| Anti-MnSOD_2_ antibody | Abcam | Ab13533 | 1:200 |
| Anti-HO-1 antibody | Abcam | Ab13248 | 1:300 |
| Substance P antibody (N-18) | Santa Cruz | SC9758 | 1:200 |
| Anti-CGRP antibody | Abcam | Ab81887 | 1:100 |
| Alexa Fluor 488 goat anti-rabbit IgG | Beyotime | A0423 | 1:500 |
| Alexa Fluor 488 goat anti-mouse IgG | Beyotime | A0428 | 1:500 |
| Cy3-loaded Donkey Anti-Goat IgG | Beyotime | A0502 | 1:500 |

**Reverse transcription quantitative-polymerase chain reaction (PCR)**

Total RNA from corneas, TG neurons, and brain were extracted using NucleospinRNA Kits (BD Biosciences, Palo Alto, CA). cDNAs were synthesized using the Primescript™ First-Strand cDNA Synthesis kit (TaKaRa, Dalian, China). Real-time PCR was carried out using SYBR® Green reagents and the Applied Biosystems 7500 Real Time PCR System (Applied Biosystems, Foster City, CA). The specific primers used are listed in Table S2. The cycling conditions were 10 sec at 95°C followed by 45 two-step cycles (15 sec at 95°C and 1 min at 60°C). The quantification data were analyzed with the Sequence Detection System (SDS) software (Applied Biosystems) using GAPDH as an internal control. The relative expression of mRNA was calculated using the established 2^−ΔΔCT^ method^7^. Briefly, the mRNA level of each target gene was normalized, first to that of GAPDH and then to its mRNA level in the NC group with corneal abrasions.

Table S2 Sequences information for PCR

| Gene name | Primer Sequences (F) | Primer Sequence (R) |
| --- | --- | --- |
| IL-1β | CTTTCCCGTGGACCTTCCA | CTCGGAGCCTGTAGTGCAGTT |
| IL-6 | ACCACTCCCAACAGACCTGTCT | CAGATTGTTTTCTGCAAGTGCAT |
| TNFα | AATGGCCTCCCTCTCATCAGT | GGCTGGCACCACTAGTTGGT |
| NF-κB | AAACCGTATGAGCCTGTGTTCAC | GACCCAACTTCTGTGCCAGAGT |
| CXCL10 | GGTCCGCTGCAACTGCAT | GGATTCAGACATCTCTGCTCATCA |
| CXCL11 | GCTCAAGGCTTCCTTATGTTCAA | AGCCTTCATAGTAACAATCACTTCAACT |
| CXCL12 | TGCATCAGTGACGGTAAACCA | GTTGTTCTTCAGCCGTGCAA |
| BDNF | GGGTCACAGCGGCAGATAAA | TGCAGCCTTCCTTGGTGTAAC |
| CNTF | GCCGCTCTATCTGGCTAGCA | TCATCTCACTCCAGCGATCAGT |
| TAC1 | GACCCAAGCCTCAGCAGTTC | CCATTAGTCCAACAAAGGAATCTGT |
| CGRPα | GCATGGCCACTCTCAGTGAA | TTCCCACACCGCTTAGATCTG |
| GAPDH | GGTGAAGGTCGGTGTGAACGGA | TGTTAGTGGGGTCTCGCTCCTG |

**Measurement of reactive oxygen species (ROS) generation and antioxidant molecule expression**

The ROS level is an important biomarker for oxidative stress, and increased levels indicate higher oxidative stress. For ROS staining in the cornea and TG neuron, fresh tissues were embedded using OCT and cut into 5-μm-thick sections. After adequate washing with PBS three times, the sections were incubated with 10 µM of a fluorescence probe—2,7-dichlorodihydrofluorescein diacetate, acetyl ester (DCFH-DA; Molecular Probes, Eugene, OR)—for 30 min in the dark. The sections were then washed with PBS three times, for 10 min each time. The tissues were observed and images were captured using a Nikon confocal microscope. MD). In all cases, comparative digital images from different samples were obtained using identical exposure time, brightness, and contrast settings. The expressions of major intracellular free radical scavengers in corneal epithelium and TG neuron, including manganese superoxide dismutase (MnSOD), catalase, NAD(P)H dehydrogenase quinone 1 (NQO1), and heme oxygenase-1(HO-1), were evaluated with immunoflurorescent staining, as described elsewhere^8^.

**Brain toxicity evaluation during the treatment**

Brain toxicity was usually assessed based on the TUNEL assay for apoptosis and inflammatory cytokines for inflammation in cortex neurons^9,10^. TUNEL staining was performed using the ApopTag® Peroxidase In Situ Apoptosis Detection Kit S7100 (Chemicon, Billerica, MA) according to the manufacturer’s instructions. Proinflammatory cytokines were analyzed using the same procedure described in “Reverse transcription quantitative-polymerase chain reaction” above.

**Nasal ciliotoxicity**

The nasal ciliotoxicity was evaluated using both, an in situ toad palate model^11^ and an in vivo mice nasal mucosa model^12^. In the toad experiment, the upper palate of toads was exposed and treated with the nanomicelle curcumin solution for 4 h (nanomicelle curcumin solution was supplemented every 30 min to ensure that the maxillary mucosa of toad was fully submerged in the test solution). The test solution was then washed away with saline, and one piece of mucosa, about 3 × 3 mm, was dissected from the palate. The mucocilia were examined with an optical microscope at 400× magnification, and the lasting time of ciliary movement was recorded. For the in vivo nasal mucocilia toxicity study in mice, the nanomicelle curcumin solution was administered six times a day to the animals’ unilateral nostril for 14 days, by following the abovementioned procedure. Physiological saline and 10 mg/ml sodium deoxycholate solution were applied as negative and positive control, respectively, for both the experiments. Finally, the mice were sacrificed, and the nasal septum with the epithelial cell membrane on each side was carefully exposed. The mucocilia were examined under a scanning electronic microscope (SEM; JSM-T300, Japan).

**SI Results**

**Nanomicelle curcumin attenuates oxidative stress in corneal epithelium and TG neuron in diabetic mice**

The effect of nanomicelle curcumin on the regulation of diabetes-induced oxidative stress in the corneal epithelium and TG neuron was evaluated by the detection of intracellular reactive oxygen species (ROS). Representative results (Fig. S1. Green staining represents ROS, and red staining represents counterstaining with PI) show a significant increase in ROS in the corneal epithelium and TG neuron in the DC group mice when compared with the NC group mice. However, a weak ROS staining of corneal epithelium was observed in the OT group. Moreover and interestingly, a much weaker ROS staining was observed in the IN group, although a strengthened effect was not observed in the OT+IN group. The observations for TG neuron were similar to those for the cornea, but here a strengthened effect was observed in the OT+IN group.


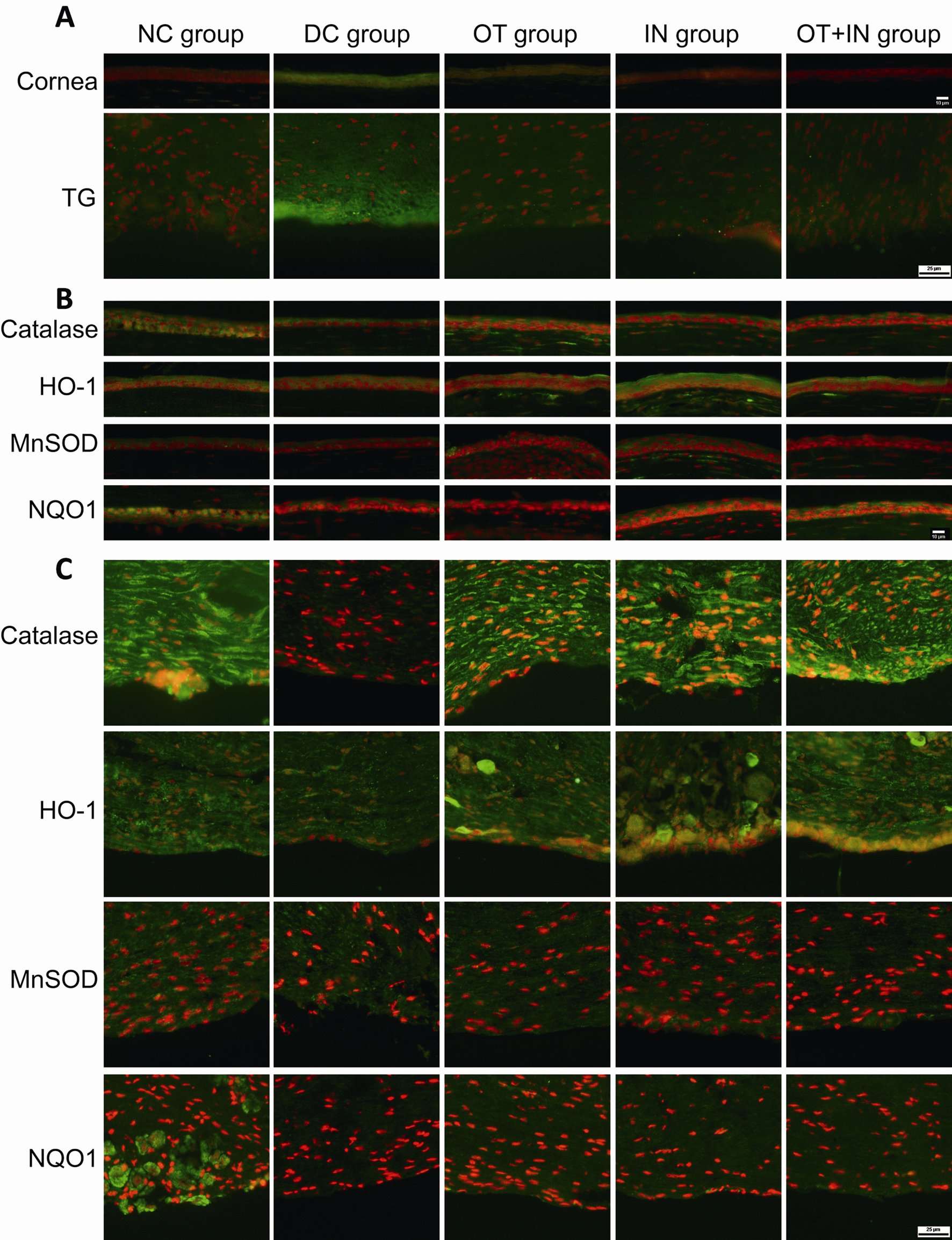


**Figure S1 Nanomicelle curcumin attenuates oxidative stress in corneal epithelium and TG neuron in diabetic mice.** Nanomicelle curcumin treatment restored the weak ROS staining, as is seen in the corneal epithelium and TG neuron (n=3) (A). Immunoflurorescent staining showed that the protein levels of NQO1, Catalase, MnSOD, and HO-1 were significantly increased in the corneal epithelium (B) and TG neuron (C) after nanomicelle curcumin treatment (n=3). Green staining represents ROS or protein level of NQO1, Catalase, MnSOD, or HO-1, and red staining represents counterstaining with PI.

In the OT group mice, the expressions of major intracellular free radical scavengers in the corneal epithelium and TG neuron, including manganese superoxide dismutase (MnSOD), catalase, NAD(P)H dehydrogenase quinone 1 (NQO1), and heme oxygenase-1(HO-1) were also recovered, as assessed by immunofluorescent staining (Fig. S1. Green staining represents protein level of NQO1, Catalase, MnSOD, or HO-1, and red staining represents counterstaining with PI). A significant recovery of these free radical scavengers was also observed in the IN group mice, although a strengthened effect was not apparent in the OT+IN group.

**TUNEL staining**

TUNEL staining was performed to define DNA damage in brain tissue in the present study. Few TUNEL-positive cells were seen in the NC and DC groups. None of the three treatment groups showed an increase in TUNEL-positive cells. These results suggest that nanomicelle curcumin does not have negative effects in normal and diabetic brain (Fig. S2).

**
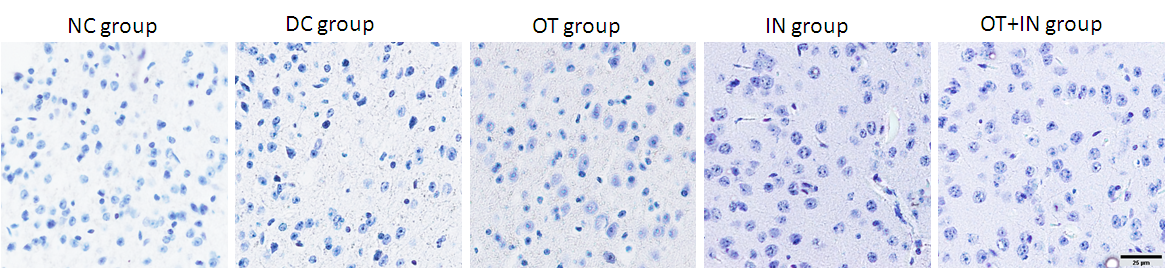
**

**Figure S2 Representative images of TUNEL staining in the cortex neurons.** TUNEL staining was performed in normal and diabetic mice tissues after corneal epithelium abrasion and 7-day nanomicelle curcumin solution treatment (n=3). Very few TUNEL-positive cells were seen in the NC group and the DC group. All three treatment groups showed no increase in TUNEL-positive cells. The bar is 25 μm.

**Nasal ciliotoxicity studies**

When developing intranasal drug delivery systems, it is important to evaluate the possible effects of a nasal formulation on beating and morphology of cilia ^13^. The in situ toad palate model has been widely used in evaluating the ciliotoxicity of intranasal formulations for its convenience and robustness, and was adopted here to evaluate the local safety of the nanomicelle curcumin solution (Fig. S3). The cilia on the mucosa were found to be intact, dense, and beat actively in the saline (negative control) and nanomicelle curcumin solution groups; however, either no cilia on the mucosa or a few exfoliated cilia were observed after treatment with 10 mg/ml deoxysodium cholate (positive control). The results exhibited the safety of the nanomicelle curcumin solution for nasal administration.

In the in vivo mice nasal mucosa model, nasal ciliotoxicity experiments by SEM were also performed to study the toxicity of the nanomicelle curcumin solution. As shown in Fig. S3, nasal mucosa with loss of epithelial cells, loss of cilia, and shrinkage of mucosal layer were observed after 2 weeks of treatment with 10 mg/ml deoxysodium cholate, but the cilia on the mucosa were intact, dense, and regular in the saline and nanomicelle curcumin solution groups. These results were in agreement with the results from the in situ toad palate model. Therefore, it can be concluded that the nanomicelle curcumin solution shows no toxicity on nasal mucosa and is safe for nasal administration.

**
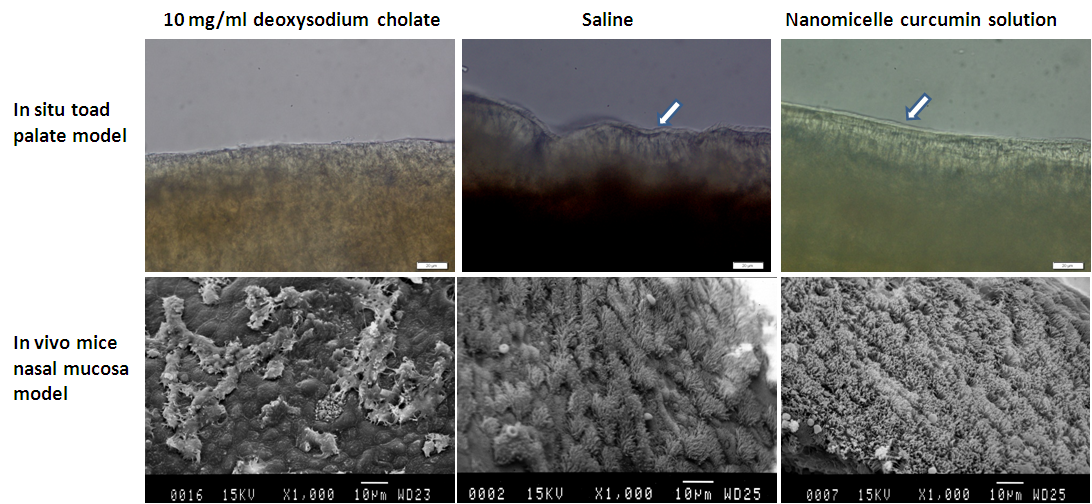
**

**Figure S3 Nanomicelle curcumin causes no nasal ciliotoxicity.** The in situ toad palate model test showed that cilia on the mucosa were intact, dense, and beat actively in the saline (negative control) and nanomicelle curcumin groups, whereas no cilia or few exfoliated cilia on the mucosa were observed after treatment with 10 mg/ml deoxysodium cholate (positive control). Cilia are indicated by the arrow (n=3). The in vivo mice nasal mucosa model showed irregular or absent cilia after 2 weeks of treatment with 10 mg/ml deoxysodium cholate, but intact, dense, regular cilia on the mucosa after treatment with saline and nanomicelle curcumin solution (n=3). Both tests confirm the safety of nanomicelle curcumin solution for intranasal administration. The bar on the in situ toad palate model images is 20 μm, and that on the in vivo mice nasal mucosa images is 10 μm.

**Reference**

1. De Rosa, R.*, et al.* Intranasal administration of nerve growth factor (NGF) rescues recognition memory deficits in AD11 anti-NGF transgenic mice. *Proceedings of the National Academy of Sciences of the United States of America* **102**, 3811-3816 (2005).

2. Guo, C.*, et al.* Nanomicelle formulation for topical delivery of cyclosporine A into the cornea: in vitro mechanism and in vivo permeation evaluation. *Sci Rep* **5**, 12968 (2015).

3. Schmidt, J.*, et al.* NSAIDs Ibuprofen, Indometacin, and Diclofenac do not interact with Farnesoid X Receptor. *Sci Rep* **5**, 14782 (2015).

4. Zochodne, D.W., Ramji, N. & Toth, C. Neuronal targeting in diabetes mellitus: a story of sensory neurons and motor neurons. *Neuroscientist* **14**, 311-318 (2008).

5. Chaudhary, S.*, et al.* Neurotrophins and nerve regeneration-associated genes are expressed in the cornea after lamellar flap surgery. *Cornea* **31**, 1460-1467 (2012).

6. Pan, Z., Fukuoka, S., Karagianni, N., Guaiquil, V.H. & Rosenblatt, M.I. Vascular endothelial growth factor promotes anatomical and functional recovery of injured peripheral nerves in the avascular cornea. *Faseb J* **27**, 2756-2767 (2013).

7. Joly Condette, C.*, et al.* Increased gut permeability and bacterial translocation after chronic chlorpyrifos exposure in rats. *PloS one* **9**, e102217 (2014).

8. Yang, L.*, et al.* Substance P promotes diabetic corneal epithelial wound healing through molecular mechanisms mediated via the neurokinin-1 receptor. *Diabetes* **63**, 4262-4274 (2014).

9. Elnaggar, Y.S., Etman, S.M., Abdelmonsif, D.A. & Abdallah, O.Y. Intranasal Piperine-Loaded Chitosan Nanoparticles as Brain-Targeted Therapy in Alzheimer's Disease: Optimization, Biological Efficacy, and Potential Toxicity. *Journal of pharmaceutical sciences* (2015).

10. Zhang, J.Z.*, et al.* Monosialotetrahexosy-1 ganglioside attenuates diabetes-enhanced brain damage after transient forebrain ischemia and suppresses phosphorylation of ERK1/2 in the rat brain. *Brain research* **1344**, 200-208 (2010).

11. Chen, J.*, et al.* Solanum tuberosum lectin-conjugated PLGA nanoparticles for nose-to-brain delivery: in vivo and in vitro evaluations. *Journal of drug targeting* **20**, 174-184 (2012).

12. Gao, X.*, et al.* Lectin-conjugated PEG-PLA nanoparticles: preparation and brain delivery after intranasal administration. *Biomaterials* **27**, 3482-3490 (2006).

13. Li, C.*, et al.* Enhancement in bioavailability of ketorolac tromethamine via intranasal in situ hydrogel based on poloxamer 407 and carrageenan. *International journal of pharmaceutics* **474**, 123-133 (2014).
